# Efficacy and Safety of Radiofrequency and Focused Ultrasound in Facial Rejuvenation: A Single‐Center, Single‐Blind, Non‐Randomized Prospective Trial

**DOI:** 10.1111/jocd.70407

**Published:** 2025-08-20

**Authors:** Boyu Zhao, Pinglu Li, Yujie Fu, Yue Quan, Huiping Wang, Leran Zhao, Xinyue Pang, Zhaoyang Liu, Lisha Tan, Jiao Peng, Wanxing Zhao, Zhongqi Jiang, Danfeng Yuan, Shuping Hou

**Affiliations:** ^1^ Department of Dermatovenereology Tianjin Medical University General Hospital/Tianjin Institute of Sexually Transmitted Disease Tianjin China

**Keywords:** efficacy, facial rejuvenation, focused ultrasound, radiofrequency, safety

## Abstract

**Background:**

Although both radiofrequency (RF) and focused ultrasound (FU) are commonly used non‐invasive skin‐tightening methods, comparative studies between the two methods are lacking.

**Objective:**

To study and compare the efficacy and safety of RF and FU in facial rejuvenation.

**Methods:**

We recruited 36 patients to receive a single treatment: the RF group (*n* = 21) and the FU group (*n* = 15). Every participant was evaluated on baseline (T0), 1‐month follow‐up (T1) and 3‐month follow‐up (T3). Three blinded dermatologists used the FLR scale, Modified Fitzpatrick Wrinkle Scale (MFWS), and Allergan Skin Roughness Scale to assess facial wrinkles. Safety profiles were recorded, and subjects completed questionnaires to provide subjective evaluations.

**Results:**

Intergroup comparison indicated that the ultrasound group showed superior efficacy over the RF group in the mid‐face (*Z* = 2.915, *p* = 0.004) and lower face (*Z* = 2.142, *p* = 0.046) on T1. Intragroup comparison observed a statistically significant reduction in FLR and MFWS scores with both treatments. Subjective satisfaction aligned with objective results, and no severe adverse reactions were observed.

**Conclusion:**

Both RF and FU are effective in facial rejuvenation, though their efficacy differs. They are comparably safe and yield high patient satisfaction.

**Trial Registration:**

ChiCTR number: ChiCTR2500097849

## 1 Introduction

Aging is a universal phenomenon in the biological realm, and facial aging has a negative impact on various aspects of human life, including physiological, psychological, and social dimensions. The growing demand to reduce wrinkles and skin laxity has led to the development of various rejuvenation methods. Among these, minimally invasive and noninvasive techniques are increasingly used for facial rejuvenation. These techniques offer several advantages, such as reduced pain and allowing for quick recovery [1].

Radiofrequency (RF) and focused ultrasound (FU) are commonly used non‐invasive methods for skin tightening that can be safely applied to any skin type [1]. Both methods target the deeper layers of the skin, causing a rise in local temperature, which produces a thermal effect that leads to collagen contraction and stimulates the production of new collagen [2].

Recent research has indicated that there is varying efficacy of RF and FU in facial rejuvenation [3, 4]. Some studies have investigated the combined use of RF and FU for facial rejuvenation [1, 5, 6], yet there is a lack of data comparing the differences in efficacy and safety between these two treatments. The aim of our study was to compare the efficacy and safety of RF and FU in facial rejuvenation.

## 2 Materials and Methods

The study recruited 36 patients aged between 25 and 55 years who had a need for facial rejuvenation. A senior dermatologist conducted the inclusion assessments. Exclusion criteria included individuals who were pregnant or breastfeeding, those with implanted cardiac pacemakers or other similar electronic devices and metal implants, patients with other significant medical conditions, a history of anti‐aging treatments within the last 6 months, individuals unable to cooperate, and those with poor adherence who could not follow up as scheduled. All participants completed the subsequent treatment and follow‐up; they received a single treatment: the RF group (*n* = 21) and the FU group (*n* = 15). Both treatment methods were approved by the institutional ethics committee (IRB2023‐YX‐016‐01). All patients were informed of the benefits, risks, and potential complications of the treatment before enrollment and signed informed consent forms. This research is in compliance with the tenets of the Declaration of Helsinki.

### 2.1 Treatment

Each participant received a single treatment of either RF or FU. Prior to treatment, a gentle cleanser was used to remove all cosmetics and skincare products, and any metal jewelry around the ears was removed. The RF group also received a topical anesthetic mask, which was covered with plastic wrap for 20 min.

The RF group utilized the Thermolift Plus (Alma, Israel). The treatment for the lower face was administered at a power range of 90–250 W, with a total energy of 110–300 kJ, an on time of 0.4–3.0 s, an off time of 1.1–1.6 s, and a pulse count of 300–400 per side. The power and duration were adjusted based on the patient's tolerance to achieve the maximum tolerable heating sensation. Depending on the different facial areas and the patient's specific conditions, four settings—S1, S2, D1, and D2—were selected, corresponding to depths of 1.5, 2.5, 3.5, and 4.5 mm, respectively, targeting the superficial dermis, deep dermis, SMAS fascia layer, and adipose layer.

The FU group used XEMIS (Simis Medical Technology (Shenzhen) Co. Ltd., China). Initially, an ultrasound probe was used to measure the SMAS layer depth in the treatment area, followed by the handheld zoom transducer set at a power of 4–5 W, delivering 30–50 pulses per subregion. Next, a circular or sliding motion was applied using the zoom head for a slightly faster treatment, with each area receiving 3–5 min of treatment. Upon completion with the zoom head, a fixed‐focus 3.0 mm head and 3.0 mm‐transducer were subsequently applied to each subregion. Power and treatment duration were adjusted based on the patient's tolerance to achieve the endpoint response.

After treatment, a room‐temperature facial mask was applied for 20 min. During the study period, each participant was advised to use sunscreen on the face (minimum SPF 30) and to avoid direct sun exposure.

### 2.2 Clinical Evaluation

Standardized photographs were taken with a Nikon D3400 digital camera at baseline (T0), 1‐month follow‐up (T1) and 3‐month follow‐up (T3) for documentation. Three blinded dermatologists assessed the mid‐face, lower face, nasolabial folds, and roughness using the FLR scale [7], Modified Fitzpatrick Wrinkle Scale (MFWS) [8] and Allergan Skin Roughness Scale (ASRS) [9]. Pain levels during the procedure were recorded using a Visual Analog Scale (VAS), ranging from 0 to 10, where 0 represents no pain and 10 represents severe, intolerable pain. Three months after the treatment, a questionnaire was administered to obtain subjective evaluations from the patients.

### 2.3 Statistical Analysis

Statistical analyses were performed using SPSS version 26.0. Continuous data were presented as quartiles or mean (standard deviation) based on normality. Categorical data were expressed as frequency and percentage. Normality of distributions was assessed using the Shapiro–Wilk test. Intergroup comparisons were conducted using the Mann–Whitney *U* test and *t*‐test, while intragroup comparisons were analyzed with the Friedman test. If significant (*p* < 0.05), post hoc pairwise comparisons were performed using the Wilcoxon test. All results with *p* < 0.05 were considered statistically significant.

## 3 Results

### 3.1 Characteristics of Participants

**FIGURE 1:**
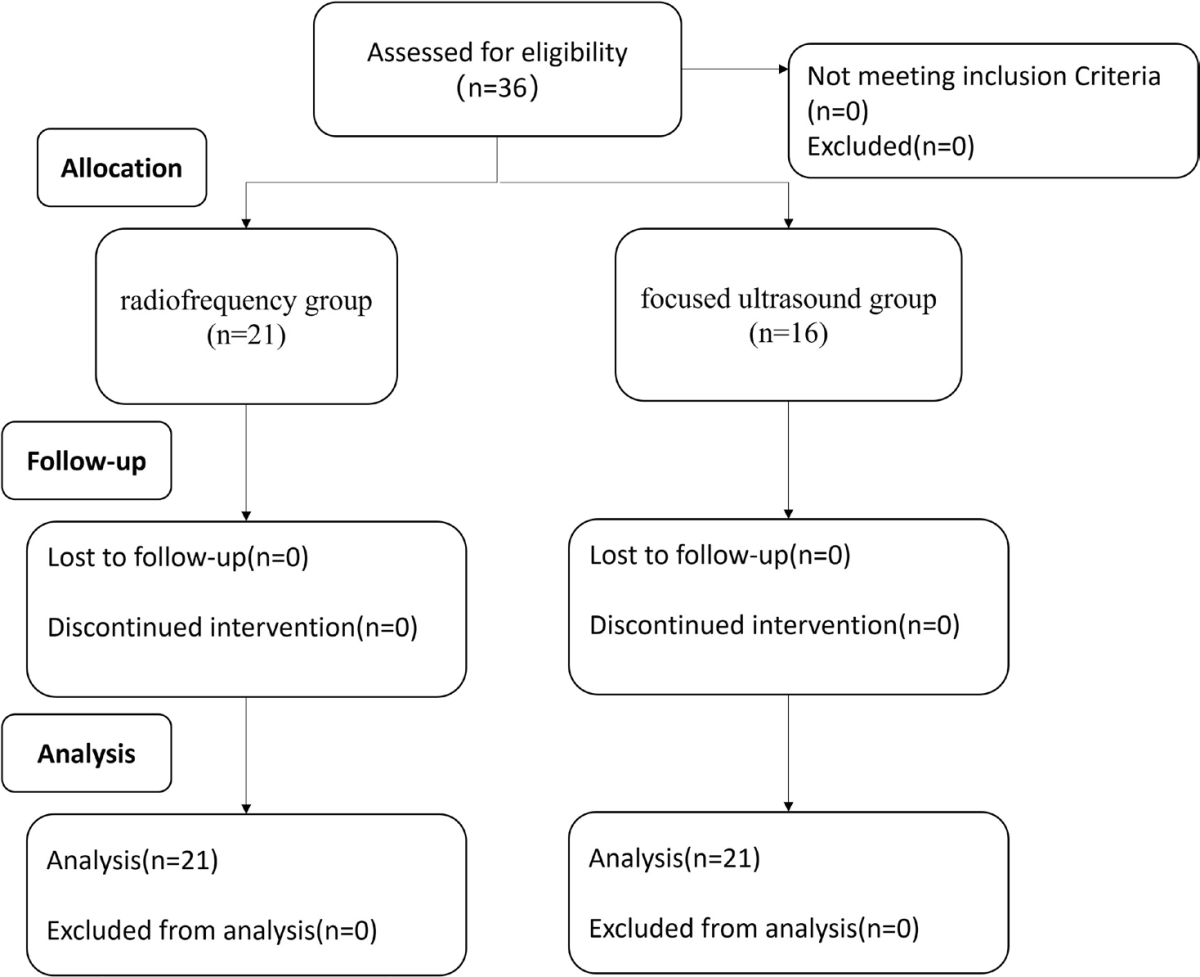
Flow diagram allocation of participants.

Thirty‐six participants were included in the beginning, and they all completed the protocols (Figure 1). The RF group consisted of 21 patients (21 females, 0 male) with a mean age of 40.57 years and a standard deviation of 6.63. The FU group included 15 patients (14 females, 1 male) with a mean age of 38.87 years and a standard deviation of 6.83. An age comparison using the *t*‐test (*t* = 0.751, *p* = 0.458) revealed no statistically significant difference. There were also no statistically significant differences between the two groups in baseline FLR scale, MWFS, or ASRS (Table 1). Baseline characteristics between the two groups were comparable.

**TABLE 1:** Characteristics of participants.

Variables	RF (*n* = 21)	FU (*n* = 15)	*Z*	*p*
FWCS	1.5 (1,2)	1.5 (1.5,2)	1.394	0.18
M‐FLR	2 (1.3)	2 (1,2)	1.019	0.34
L‐FLR	2 (1,3)	2 (1,3)	0.253	0.825
ASRS	0 (0,1)	0 (0,1)	0.659	0.059

*Note:* Characteristics of participants between two groups revealed no statistically significant difference.

Abbreviations: ASRS = Allergan Skin Roughness Scale, FU = focused ultrasound, L‐FLR = low‐face FLR scale, M‐FLR = mid‐face FLR scale, MFWS = Modified Fitzpatrick Wrinkle Scale, *n* = sample number, RF = radiofrequency.

* Significance level *p* < 0.05.

### 3.2 Clinical Outcome Assessment

**FIGURE 2:**
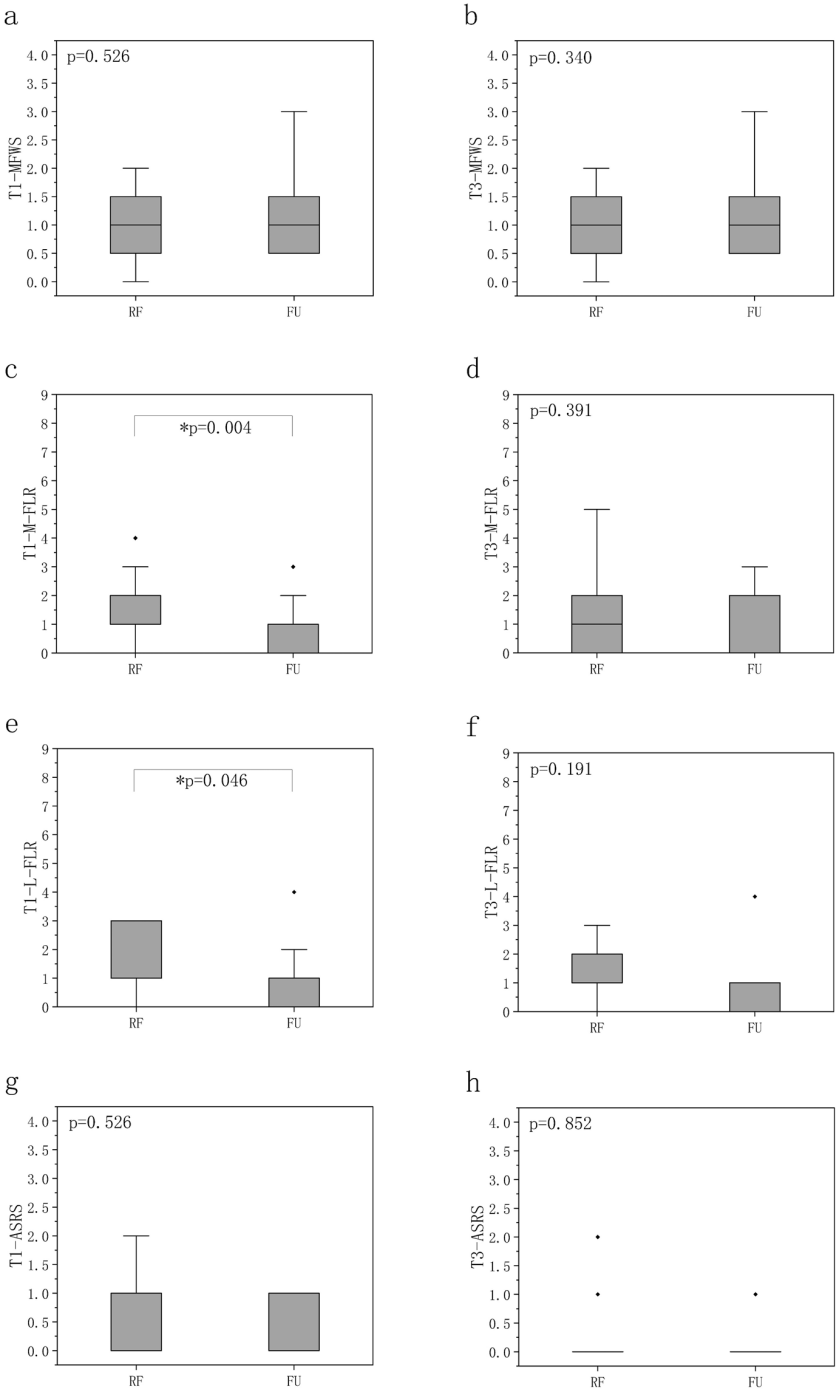
Intergroup comparison of FLR scale, MFWS, and ASRS. ASRS = Allergan Skin Roughness Scale, FU = focused ultrasound, L‐FLR = low‐face FLR scale, M‐FLR = mid‐face FLR scale, MFWS = Modified Fitzpatrick Wrinkle Scale, RF = radiofrequency, T1 = 1‐month follow‐up, T3 = 3‐month follow‐up. Mann–Whitney *U* test; *Significance level *p* < 0.05.

**FIGURE 3:**
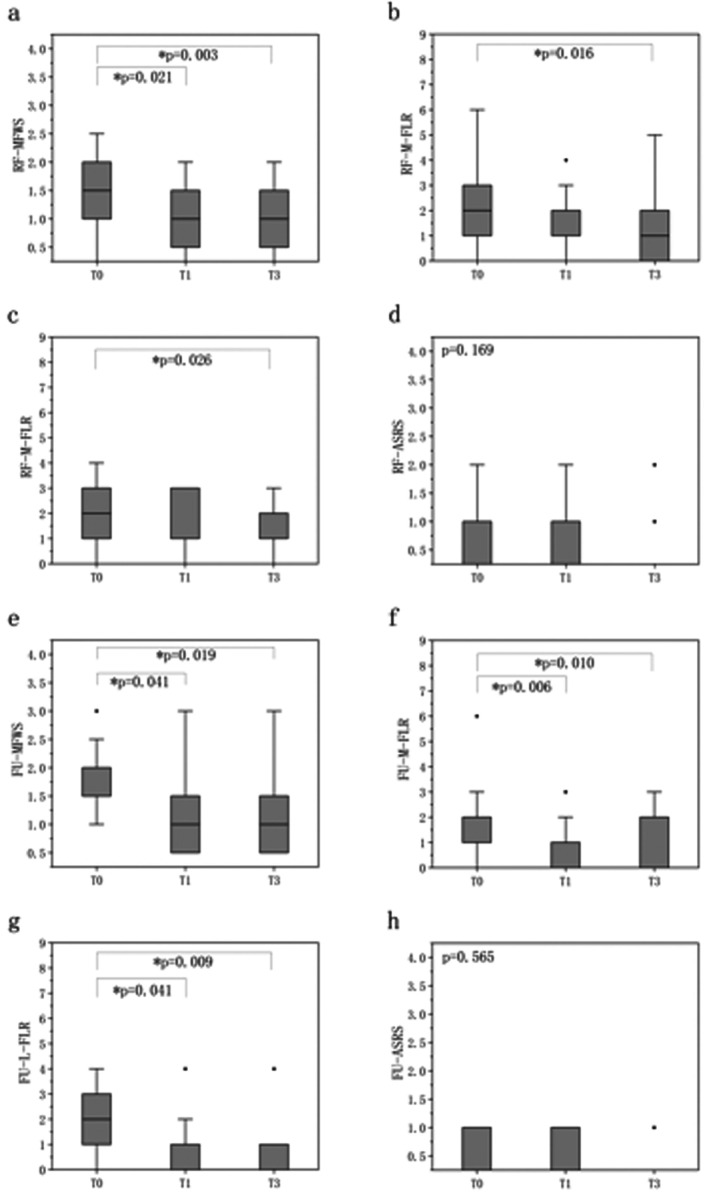
Intragroup comparison of FLR scale, MFWS, and ASRS. ASRS = Allergan Skin Roughness Scale, FU = focused ultrasound, L‐FLR = low‐face FLR scale, M‐FLR = mid‐face FLR scale, MFWS = Modified Fitzpatrick Wrinkle Scale, RF = radiofrequency, T0 = initial assessment, T1 = 1‐month follow‐up, T3 = 3‐month follow‐up. Friedman test; *Significance level *p* < 0.05.

Intergroup comparison indicated that the ultrasound group showed superior efficacy over the RF group in the mid‐face (*Z* = 2.915, *p* = 0.004) and lower face (*Z* = 2.142, *p* = 0.046) on T1, with no significant differences observed in nasolabial fold and roughness (Figure 2). Intragroup comparison demonstrated statistically significant improvements in the RF group in MFWS on T1 and T3, in mid‐face and lower face on T3 (*p* < 0.05). In the ultrasound group, significant improvements were observed in wrinkle scores of the nasolabial fold, mid‐face, and lower face on both T1 and T3 (*p* < 0.05) (Figure 3).

### 3.3 Questionnaire Results

**FIGURE 4:**
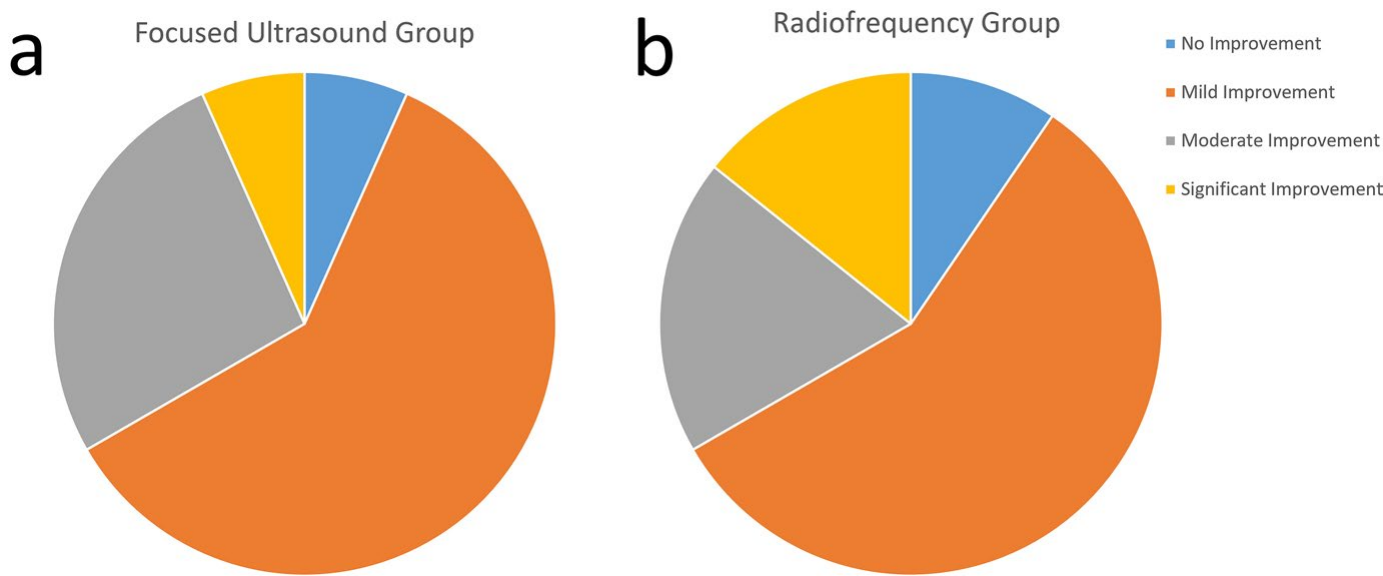
Distribution of patients according to perceived improvement.

In the RF group, 90.4% of patients believed their facial condition had improved, with 57.1% (12/21) reporting mild improvement, 19.0% (4/21) reporting moderate improvement, and 14.3% (3/21) reporting significant improvement. In the FU group, 93.3% of patients felt their facial condition had improved, with 60.0% (9/15) reporting mild improvement, 26.7% (4/15) reporting moderate improvement, and 6.7% (1/15) reporting significant improvement (Figure 4).

**FIGURE 5:**
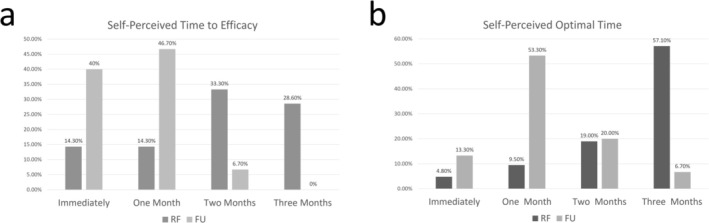
Subjective evaluation. FU = focused ultrasound, RF = radiofrequency.

In the RF group, 33.3% (7/21) of patients reported noticeable effects at 2 months post‐treatment, while 57.1% (12/21) felt that the treatment was most effective at 3 months post‐treatment. In the FU group, 40% (6/15) of patients observed initial effects immediately after treatment, and 46.7% (7/15) noticed effects at 1 month post‐treatment; 53.3% (8/15) of patients believed the optimal time for efficacy was at 1 month (Figure 5).

### 3.4 Safety Profiles

The mean pain score in the RF group was 4.62, with a standard deviation of 1.99. In the FU group, the mean pain score was 4.20, with a standard deviation of 1.70. Both groups did not experience severe pain scores of 8–10. All patients in both the RF and FU groups experienced mild edema and mild to moderate erythema as the endpoint responses, which resolved within half an hour to 3 days. In the FU group, 6 patients (40.0%) experienced bilateral mandibular or zygomatic pain and tenderness, which resolved approximately 3–30 days post‐treatment. One patient (6.67%) experienced tooth sensitivity, which resolved after 7 days. Another patient (6.67%) had facial epidermal damage, which healed within a week. No patients in either group developed scarring, pigmentation, or other complications.

## 4 Discussion

In this study, we analyzed the efficacy of RF and FU for facial rejuvenation, assessing and comparing scores for the nasolabial folds, mid‐face, lower face, and skin roughness at baseline, 1‐month follow‐up, and 3‐month follow‐up. To our knowledge, while there are reports that combining RF and FU may enhance efficacy [6], there is a lack of clinical studies directly comparing the effectiveness of these two methods. Although Kim et al. demonstrated histologically that the thermal responses induced by FU and RF are indistinguishable [5], this study is the first to clinically compare the efficacy differences between RF and FU.

The pursuit of facial rejuvenation is a universal goal. RF and FU are non‐invasive methods applied to this end. Both work by inducing thermal effects, leading to the temporary contraction of collagen, which then triggers collagen remodeling and stimulates the synthesis of new collagen and elastin. This process is accompanied by the necrolysis of adipose tissue, effectively addressing facial skin laxity and aging [2, 10, 11, 12].

Reports on RF and FU for facial rejuvenation have been positive. Nelson et al. [13] treated 14 patients with facial and neck laxity using the Forma bipolar RF device, with all patients (14/14) showing clinical improvement and 21% (3/14) experiencing significant improvement 4–6 weeks post‐treatment. Pereira et al. [14] used the Límine bipolar RF device, reporting a reduction in MFWS in 16 patients 8 weeks after treatment (*p* = 0.041). In 2023, a study using the Modula bipolar RF device observed increased overall skin hydration and elasticity in 62 patients' post‐treatment [15]. Aşiran Serdar et al. [16] used the Doublo‐S high‐intensity FU device (Hironic Co. Ltd.) with 3.0 and 4.5 mm depth probes to treat 75 patients, achieving an improvement rate of over 80% in the jawline after 3 months. In our study, both RF and FU effectively improved wrinkle scores in the nasolabial folds, mid‐face, and lower face. We observed that only a small portion of patients experienced improvements in skin texture, with no statistically significant differences in overall ASRS post‐treatment; this finding aligns with the results reported by Hwang et al. [17]. RF and FU primarily target the dermis and the superficial muscular aponeurotic system (SMAS) [2], with only a mild effect on the epidermis. This limited epidermal action could be the reason why most patients did not experience noticeable improvements in skin texture. The efficacy in the mid‐face (*Z* = 2.915, *p* = 0.004) and lower face (*Z* = 2.142, *p* = 0.046) was superior in the FU group compared to the RF group on T1. However, there was no significant difference in efficacy between the two methods in the mid‐face and lower face on T3. Subjective survey results also indicated that the FU group patients perceived treatment effects immediately after the procedure, whereas the RF group patients reported noticeable effects starting from 1 or 2 months post‐treatment. This may suggest that the FU group offers a faster onset of effects compared to the RF group, providing earlier visible improvement. Regarding other indicators of facial rejuvenation, the two treatments were comparable in effectiveness. These findings help fill a current gap in research on this topic.

Pain is a subjective experience, with varying thresholds and tolerance levels among individuals. Evidence suggests that RF treatment typically exhibits mild to moderate pain levels [18, 19]. Felipe et al. [20] conducted a retrospective study on 290 patients treated with Thermage, finding that 11.49% reported significant pain, while 2.7% experienced second‐degree burns, and 1.22% developed persistent erythema. In our study of 21 patients in the RF group, despite the use of a topical anesthetic mask to mitigate pain before treatment, the average VAS score for pain was 4.62, with 23.81% (5/21) reporting scores of 7 or higher. In the FU group, where no anesthetic mask was used, 13.3% (2/15) reported pain scores of 7 or higher. This suggests that patients may have slightly better tolerance for FU treatment compared to RF. Stronger local anesthetics or nonsteroidal medications may alleviate pain and improve patient acceptance during the procedure. A 2024 study showed that combining buccal and superficial cervical nerve blocks could enhance comfort in facial and neck rejuvenation treatments [21]. However, RF treatments ideally apply the maximum tolerable energy, and fully eliminating pain could impair the practitioner's judgment, potentially leading to excessive heat accumulation and severe burns. In our study, one patient (6.67%) experienced tooth sensitivity post‐RF treatment, which resolved within a week. Nervous system complications with FU are rarely reported. Suh et al. [22] studied 22 patients treated for facial laxity with FU (Ulthera LLC, Mesa, AZ); one patient experienced mild perioral numbness, which resolved within a month. The exact mechanism behind nerve damage following treatment is not yet clear but may involve mandibular nerve branch injury due to thermal effects. Harris et al.'s [23] research using FU for lower face and neck treatments noted that the use of a superficial 7‐MHz, 3.0‐mm transducer was more likely to cause adverse effects, as higher‐frequency transducers pose an increased risk of tissue damage. In our study, one patient (6.67%) experienced superficial facial epidermal damage, which healed within a week. This may have been due to shallow treatment or improper handling during the procedure.

The limitations of this study include a small sample size and the absence of histopathological evidence, which may affect the accuracy of the results. However, our findings do support certain previous clinical observations.

## 5 Conclusion

In summary, RF and FU therapies are two widely used methods in facial and neck rejuvenation. Our study demonstrates significant improvements in wrinkle reduction along the mid‐face, lower face, and nasolabial folds with both treatments. While efficacy differences exist, FU shows a faster onset of effect compared to RF; though more data are needed to substantiate this finding. Patients experienced mild to moderate pain during treatment, with no reports of severe pain. Adverse reactions were minimal with RF, whereas localized pain and tenderness were common with FU but resolved spontaneously. For patients who are unwilling or contraindicated for surgery, RF or FU offer safe, effective, non‐invasive, and non‐destructive options for facial rejuvenation.

## Author Contributions

Pinglu Li: writing – original draft, validation, formal analysis, visualization, data curation, methodology. Boyu Zhao: writing – original draft, validation, formal analysis, visualization, data curation. Yujie Fu: investigation, formal analysis, validation, software. Yue Quan: methodology, validation, software. Huiping Wang: supervision, funding acquisition. Leran Zhao: investigation, validation. Xinyue Pang: investigation, software. Zhaoyang Liu: investigation, validation. Lisha Tan: resources, supervision. Jiao Peng: resources, supervision. Wanxing Zhao: resources, supervision. Zhongqi Jiang: investigation, validation, software. Danfeng Yuan: investigation, validation, software. Shuping Hou: methodology, writing – review and editing, formal analysis, funding acquisition, resources, supervision, project administration.

## Ethics Statement

The study was conducted in accordance with the Declaration of Helsinki and the International Conference on Harmonisation Guidance for Good Clinical Practice. Ethical approvals were obtained from the relevant institutional review boards at participating site (Tianjin Medical University General Hospital Medical Ethics Committee). All patients provided written informed consent in accordance with local requirements and agreed to the publication of the photos taken during the study.

## Conflicts of Interest

The authors declare no conflicts of interest.

## Supporting information


**Figure S1:** Female, 49 years, in the RF group. (a) Frontal view before procedure. (b) Frontal view 1‐month follow‐up with improvement of nasolabial fold. (c) Frontal view 3‐month follow‐up with improvement of nasolabial fold.

**Figure S2:** Female, 51 years, in the RF group.
(a) Frontal view before procedure. (b) Frontal view 1‐month follow‐up with improvement of nasolabial fold and nasojugal groove. (c) Frontal view 3‐month follow‐up with improvement of nasolabial fold and nasojugal groove.

**Figure S3:** Female, 39 years, in the RF group.
(a) Lateral view before procedure. (b) Lateral view 1‐month follow‐up with improvement of jawline sagging. (c) Lateral view 3‐month follow‐up with improvement of jawline sagging.

**Figure S4:** Female, 30 years, in the FU group. (a) Frontal view before procedure. (b) Frontal view 1‐month follow‐up with improvement of nasolabial fold and nasojugal groove. (c) Frontal view 3‐month follow‐up with improvement of nasolabial fold and nasojugal groove.

**Figure S5:** Female, 31 years, in the FU group. (a) Frontal view before procedure. (b) Frontal view 1‐month follow‐up with improvement of nasolabial fold and nasojugal groove. (c) Frontal view 3‐month follow‐up with improvement of nasolabial fold and nasojugal groove.

**Figure S6:** Female, 42 years, in the FU group. (a) Lateral view before procedure. (b) Lateral view 1‐month follow‐up with improvement of jawline sagging. (c) Lateral view 3‐month follow‐up with improvement of jawline sagging.

## Data Availability

The data that support the findings of this study are available from the corresponding author upon reasonable request.
