# Supplementary figures and images for: Efficacy and Safety of Radiofrequency and Focused Ultrasound in Facial Rejuvenation: A Single‐Center, Single‐Blind, Non‐Randomized Prospective Trial
Source: J Cosmet Dermatol. 2025 Aug 20;24(8):e70407. doi: 10.1111/jocd.70407 (PMC12365734; doi:10.1111/jocd.70407)

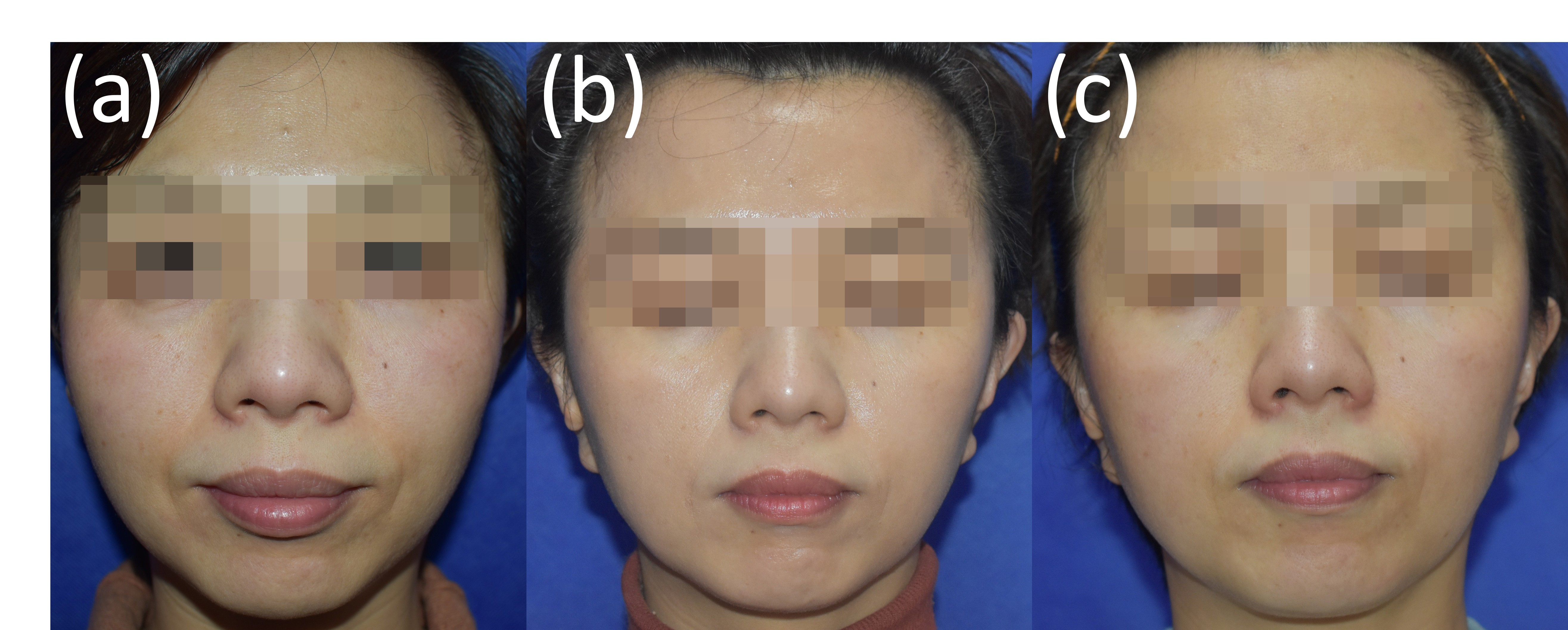

Supplement: Supplementary file 1 — Figure S1: Female, 49 years, in the RF group. (a) Frontal view before procedure. (b) Frontal view 1‐month follow‐up with improvement of nasolabial fold. (c) Frontal view 3‐month follow‐up with improvement of nasolabial fold. [file JOCD-24-e70407-s006.jpg]

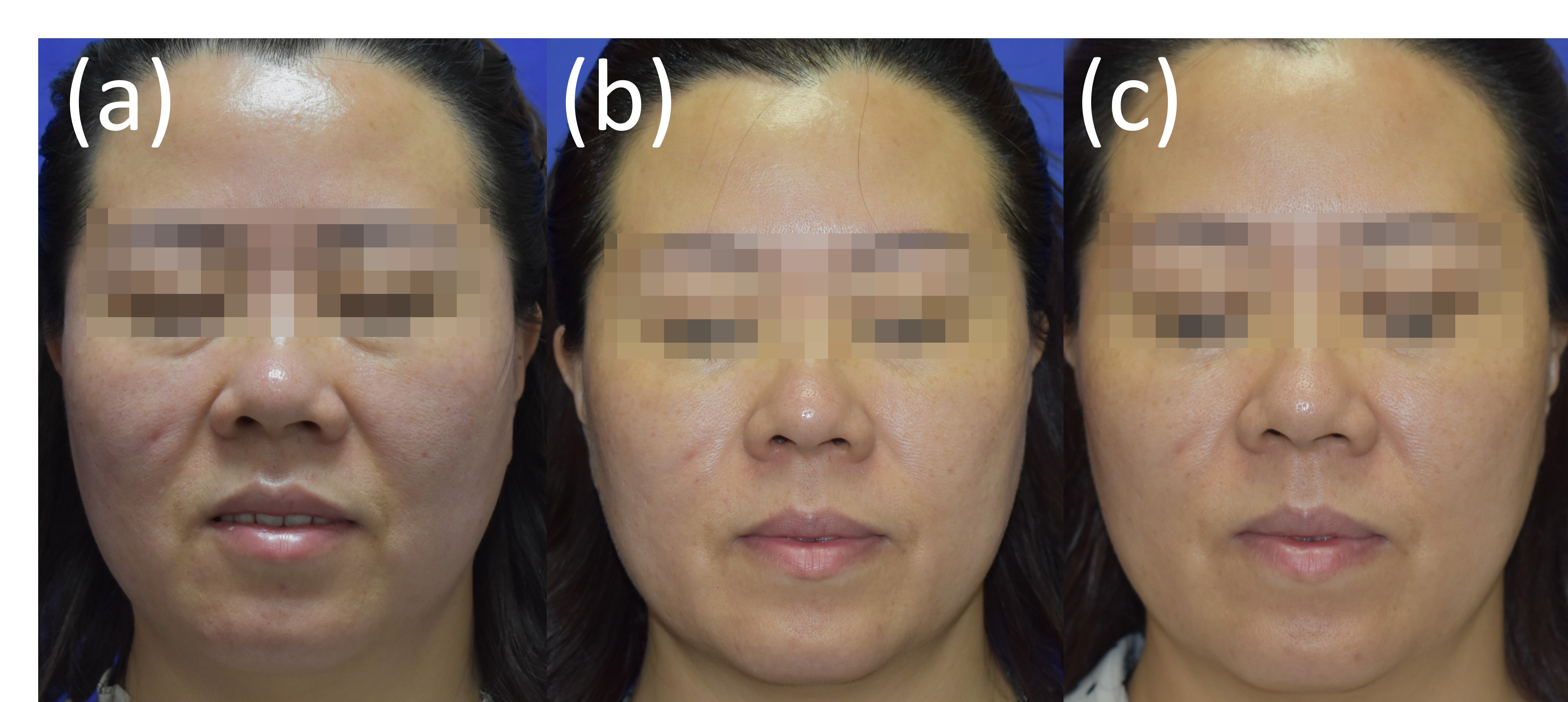

Supplement: Supplementary file 2 — Figure S2: Female, 51 years, in the RF group. (a) Frontal view before procedure. (b) Frontal view 1‐month follow‐up with improvement of nasolabial fold and nasojugal groove. (c) Frontal view 3‐month follow‐up with improvement of nasolabial fold and nasojugal groove. [file JOCD-24-e70407-s002.jpg]

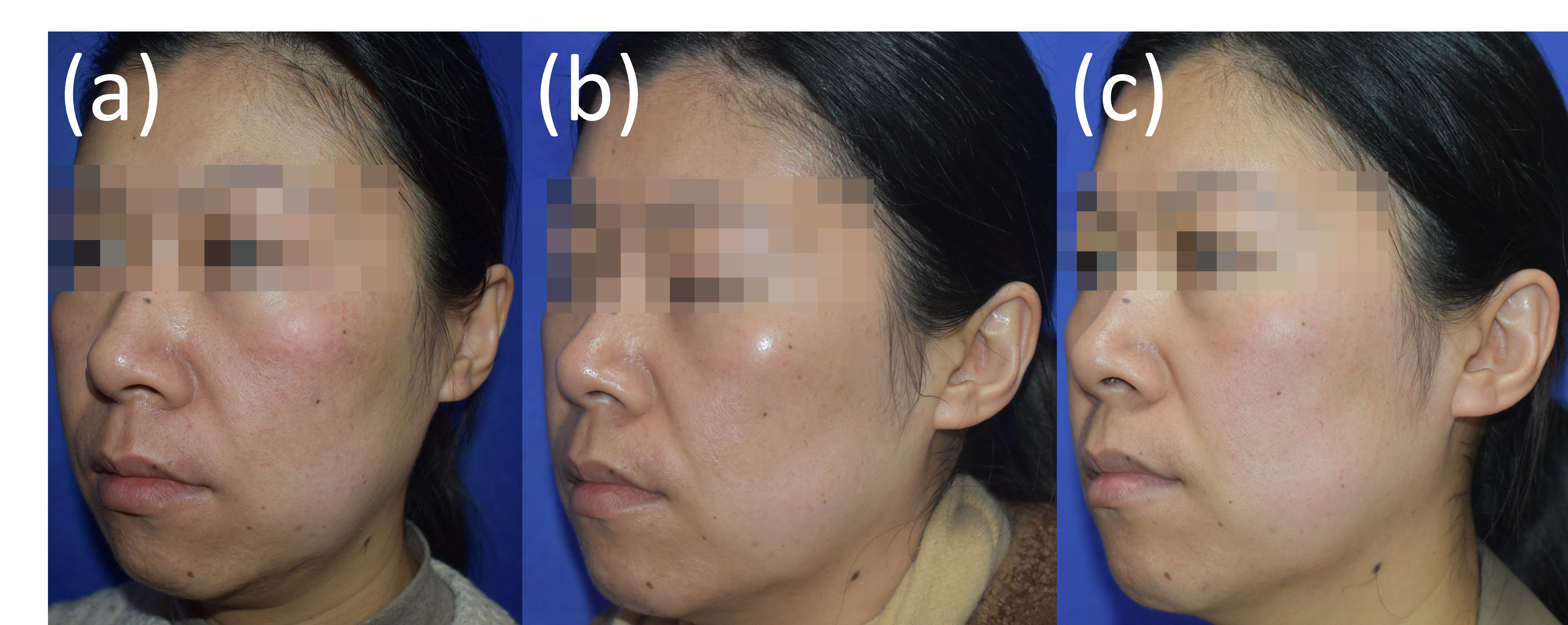

Supplement: Supplementary file 3 — Figure S3: Female, 39 years, in the RF group. (a) Lateral view before procedure. (b) Lateral view 1‐month follow‐up with improvement of jawline sagging. (c) Lateral view 3‐month follow‐up with improvement of jawline sagging. [file JOCD-24-e70407-s004.jpg]

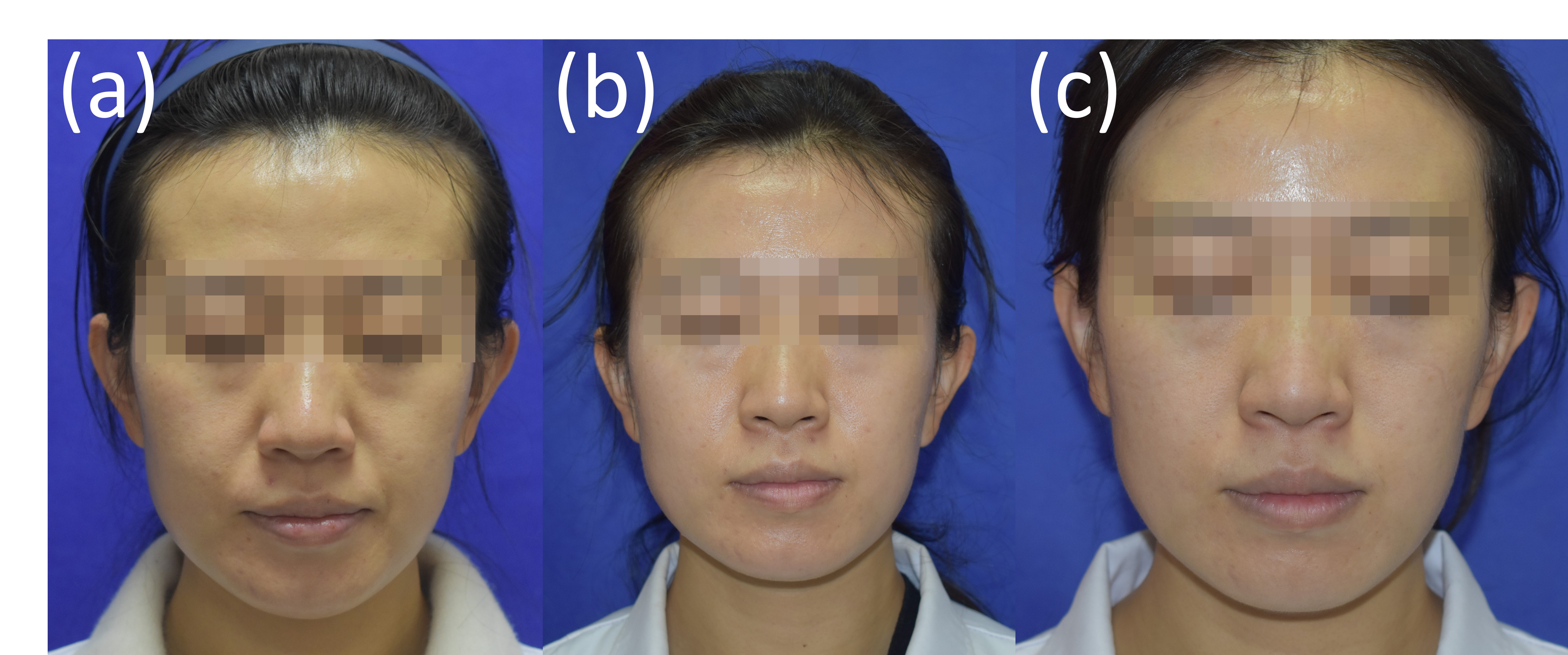

Supplement: Supplementary file 4 — Figure S4: Female, 30 years, in the FU group. (a) Frontal view before procedure. (b) Frontal view 1‐month follow‐up with improvement of nasolabial fold and nasojugal groove. (c) Frontal view 3‐month follow‐up with improvement of nasolabial fold and nasojugal groove. [file JOCD-24-e70407-s003.jpg]

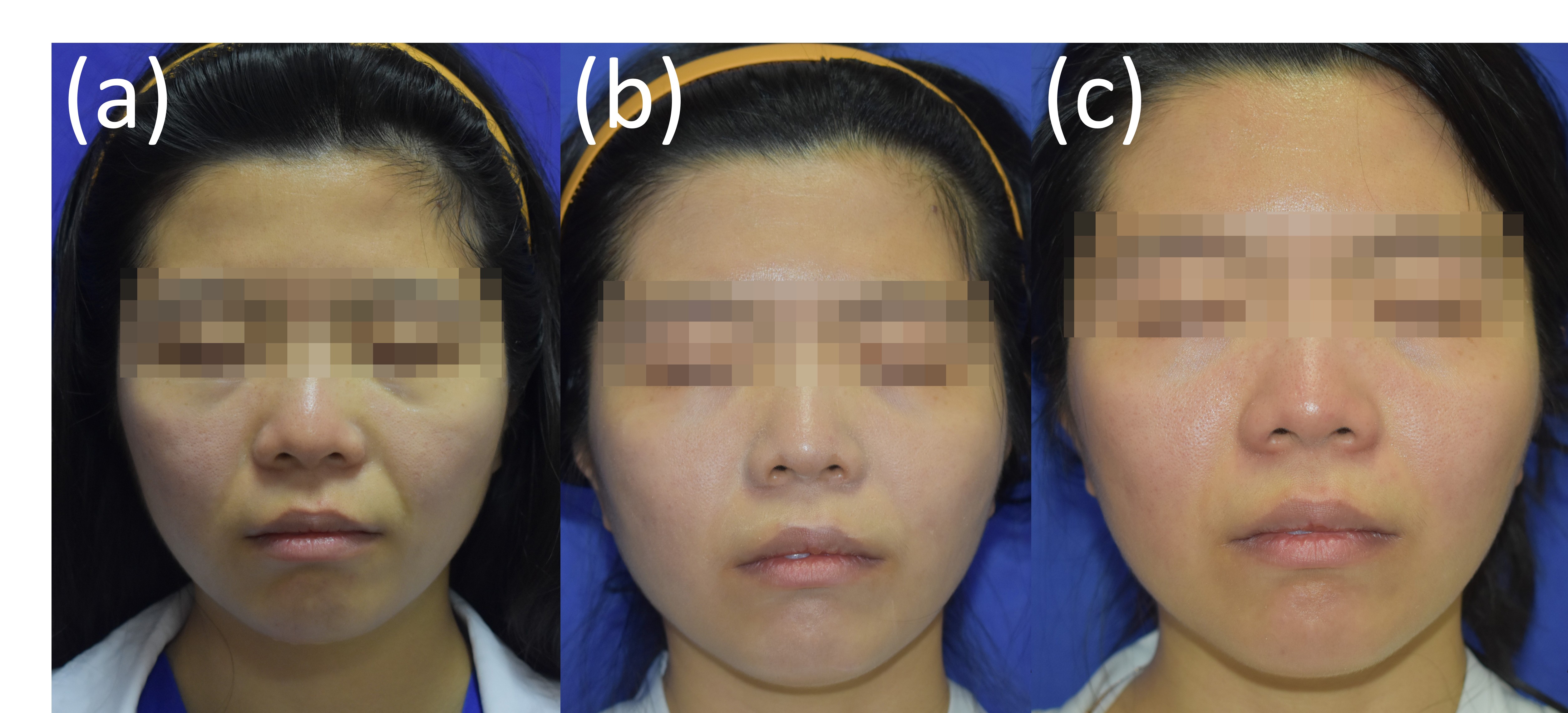

Supplement: Supplementary file 5 — Figure S5: Female, 31 years, in the FU group. (a) Frontal view before procedure. (b) Frontal view 1‐month follow‐up with improvement of nasolabial fold and nasojugal groove. (c) Frontal view 3‐month follow‐up with improvement of nasolabial fold and nasojugal groove. [file JOCD-24-e70407-s001.jpg]

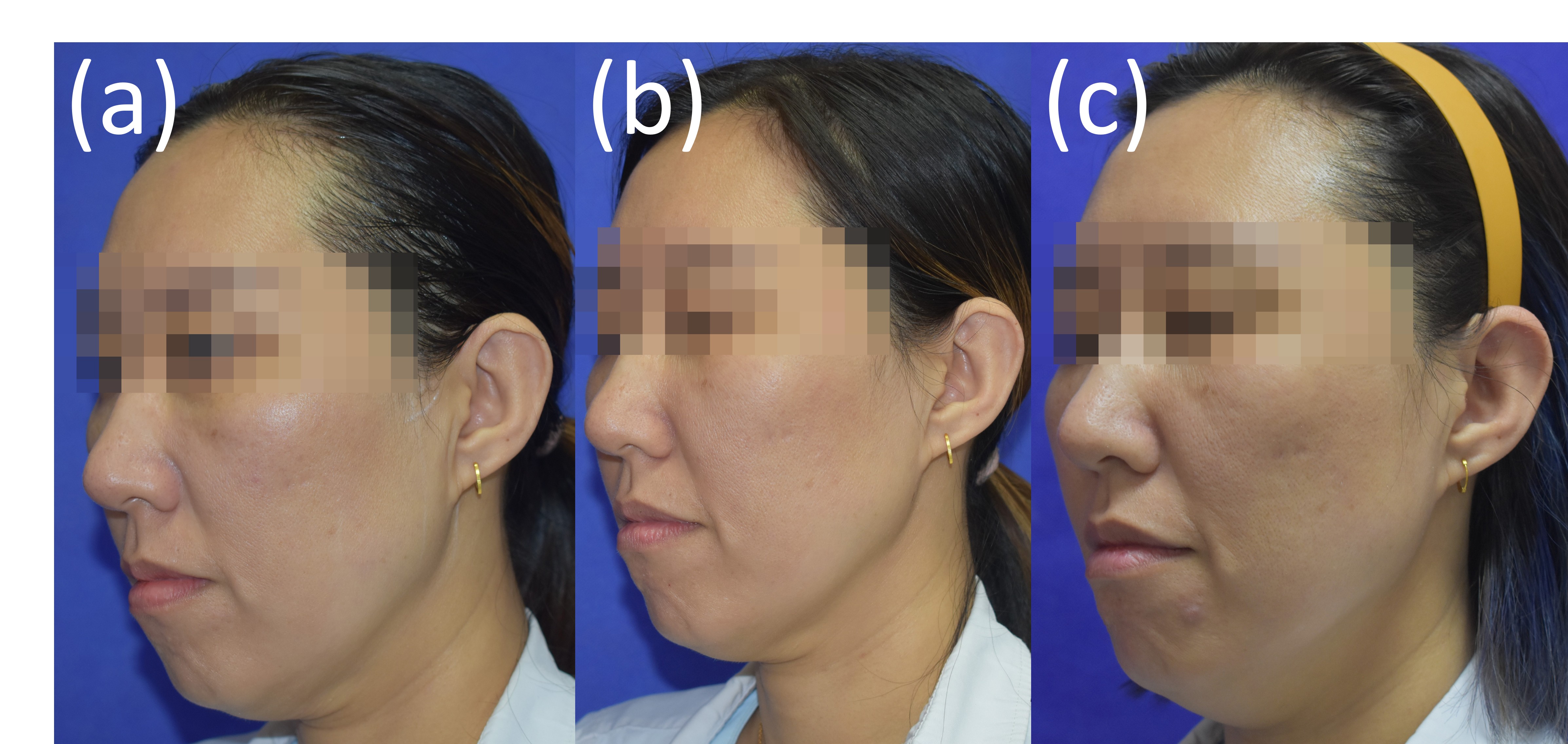

Supplement: Supplementary file 6 — Figure S6: Female, 42 years, in the FU group. (a) Lateral view before procedure. (b) Lateral view 1‐month follow‐up with improvement of jawline sagging. (c) Lateral view 3‐month follow‐up with improvement of jawline sagging. [file JOCD-24-e70407-s005.jpg]
